# Protocol for Evaluating Remote Patient Blood Pressure Monitoring Adapted to Black Women and Birthing Persons

**DOI:** 10.3390/ijerph21050603

**Published:** 2024-05-08

**Authors:** Loral Patchen, Asli McCullers, Serenity G. Budd, H. Joseph Blumenthal, W. Douglas Evans

**Affiliations:** 1 MedStar Health Research Institute, Hyattsville, MD 20782, USA; asli.mccullers@medstar.net (A.M.); serenity.g.budd@medstar.net (S.G.B.); joseph.blumenthal@medstar.net (H.J.B.); 2 Milken Institute School of Public Health, George Washington University, Washington, DC 20037, USA; wdevans@gwu.edu

**Keywords:** pregnancy, United States, hypertension, pregnancy-induced, maternal death, cardiovascular diseases, digital health, social identification, maternal health services, hospitals, urban

## Abstract

Cardiovascular disease is the leading cause of maternal death among Black women in the United States. A large, urban hospital adopted remote patient blood pressure monitoring (RBPM) to increase blood pressure monitoring and improve the management of hypertensive disorders of pregnancy (HDP) by reducing the time to diagnosis of HDP. The digital platform integrates with the electronic health record (EHR), automatically inputting RBPM readings to the patients’ chart; communicating elevated blood pressure values to the healthcare team; and offers a partial offset of the cost through insurance plans. It also allows for customization of the blood pressure values that prompt follow-up to the patient’s risk category. This paper describes a protocol for evaluating its impact. Objective 1 is to measure the effect of the digitally supported RBPM on the time to diagnosis of HDP. Objective 2 is to test the effect of cultural tailoring to Black participants. The ability to tailor digital content provides the opportunity to test the added value of promoting social identification with the intervention, which may help achieve equity in severe maternal morbidity events related to HDP. Evaluation of this intervention will contribute to the growing literature on digital health interventions to improve maternity care in the United States.

## 1. Introduction

Maternal mortality and severe morbidity events are life-altering tragedies that continue to increase in the United States (US). Maternal mortality rates were 32.9 deaths per 100,000 live births in 2021, compared with a rate of 23.8 in 2020 and 20.1 in 2019 [1]. Pregnancy complications remain the leading cause of death for women aged 20–44, yet another indicator of the sweeping impact of maternal mortality in the US [2,3]. These trends are especially concerning given the United States’ standing as a high-income country. Similar-income countries, such as Canada and the United Kingdom, not only have significantly lower maternal mortality rates but have also seen pronounced decreases within the same time frame [4,5]. 

A critical element in discussions on maternal mortality trends is the prevalent intertwining of social injustice. Consistently, Black and African American (AA) women have been disproportionately impacted by maternal mortality. In 2021, the maternal mortality rate for AA women was 69.9 per 100,000 births, three times the rate when compared to white women [1,6]. Black women frequently report feeling disempowered and discriminated against by their providers during their pregnancies and are more likely to lack access to quality, culturally competent obstetrical and gynecological care than their other-race counterparts [7,8,9,10]. With even Black celebrities and elite athletes unable to escape this profound health crisis [11,12], the burden of maternal mortality among Black birthing persons is immense. Co-existing with maternal mortality trends is the devastating prevalence of cardiovascular disease in the AA community, with nearly 60% of Black women aged 20 and over having a cardiovascular diagnosis [13]. Black women have significantly higher odds of entering pregnancy with chronic hypertension when compared to their white counterparts, and a considerable volume of Black maternal deaths are attributable to hypertension [14,15]. In synergy, maternal mortality and cardiovascular disease disparities speak to the overarching issue of structural racism’s embedment in the US healthcare system, which holds a long, dark history of harming racially marginalized persons [16,17]. 

Innovative interventions are needed to address maternal mortality health inequities, with a specific focus on the impact of comorbid cardiovascular conditions. A growing body of evidence has shown that digital interventions are an evolving strategy capable of promoting optimal maternal mortality. Presently, those of reproductive age (15–49) [18] are particularly likely to have familiarity with digital tools such as smartphones, computers, tablets, and other devices, which may help facilitate strong patient engagement, autonomy, and improved monitoring of health parameters [18,19]. Previous studies have demonstrated the efficacy of digital interventions such as smartphone applications, online platforms, and chatbots in targeting the perinatal diet, physical activity, and weight gain, as well as overall management of wellbeing during pregnancy [20,21,22]. However, while research supports the use of digital interventions to manage cardiovascular disease [23,24,25,26], the evidence to guide its adoption to address hypertensive disorders of pregnancy (HDP) and cardiovascular disease among pregnant and postpartum women remains insufficient. Even less is understood about how inequity in the digital space impacts marginalized birthing persons. Another “digital divide” has begun to emerge, with both racially marginalized and low-socioeconomic-status individuals being significantly less likely to have access to the resources necessary to fully engage with the contemporary explosion of digital health innovation [27]. The opportunity is twofold: promoting the adoption of digital tools to improve the clinical management of hypertension and HDP overall; and tailoring interventions to promote a greater improvement among racially marginalized persons, to address inequity. 

Our large, integrated health network partnered with a philanthropic organization to sponsor an initiative to improve maternal outcomes, with a focus on addressing racial inequities [28]. Grounded in the life course framework [29], this initiative highlights individual, family, social, and environmental drivers of health to reduce maternal mortality, severe maternal morbidity, and the equity gap between Black and white birthing individuals. To achieve this goal, our team will target improvements in the management of HDP by investing in digital technology with remote patient blood pressure monitoring (RBPM). RBPM refers to patients taking their own blood pressure in the home environment to monitor for elevation. 

We predict that the adoption of an integrated digital technology platform utilizing RBPM will reduce the time to diagnosis of HDP for all users. A positive association between RBPM and personal agency with respect to care during pregnancy has already been established, which suggests RBPM to be a powerful tool in addressing adverse outcomes for diverse patients [30,31,32]. While users of all backgrounds may potentially benefit from this intervention, the large disparity gaps that disproportionately impact Black birthing individuals necessitate a further step to meaningfully promote health equity. This intervention will be culturally tailored, including imagery, language, and customizations designed to foster social identification with the experience. Introducing this form of tailoring is expected to increase the engagement of Black birthing persons, and thus increase the overall engagement with and effectiveness of the intervention, to promote equity. 

These hypotheses are supported by the theory of planned behavior and social identity theory. Widely adopted in public health as a framework for understanding intentions, the theory of planned behavior is a successful framework for predicting engagement, execution, and adherence with the constellation of behaviors needed to manage blood pressure [33]. Haslam and colleagues [34] describe the impact of identity formation on health across the five domains of “(a) symptom appraisal and response, (b) health-related norms and behavior, (c) social support, (d) coping, and (e) clinical outcomes”.

Social identification is a process by which interventions are designed to promote benefits, increase engagement, and to create a sense of personal connection and meaning for participants. This approach has also been linked to the social marketing and branding of interventions, the benefits of the behavior change, and the cultural relevance of the interventions [35,36]. This approach has been successfully applied to increase the adoption of specific behaviors and to act as a mediator of behavior change in many different subject areas, including maternal health [37]. We hypothesize that cultural tailoring for the RBPM intervention through social identification will help to reduce the time to diagnosis of Black HDP users and reduce the disparity gap. 

The objectives of this article are twofold. One, to describe the RBPM intervention and its initial integration as part of an organizational initiative to improve maternity outcomes and narrow the equity gap between Black and white birthing individuals within a large, urban hospital-based obstetrical service. Two, to describe the planned evaluation protocol. To that end, we describe the technology, care integration, and planned methodology to assess its impact. All planned study activities will be conducted with approval from and under the oversight of the health system’s scientific institutional review board.

## 2. Proposed Methods

### 2.1. Conceptual Model and Approach

The selected digital technology with RBPM improves the availability of key biometric values for expectant and birthing individuals who face barriers to care and have trouble navigating healthcare systems. Patients using the app send blood pressure readings to a digital platform integrated with their electronic health record (EHR), which increases the cadence of blood pressure reading and monitoring, and offers the potential to identify HDP earlier. The digital platform also provides a library of educational resources and media to the user. The app is designed to increase patient engagement with care, reduce access barriers, and facilitate patient–physician/patient–midwife connectivity between prenatal office visits. 

**Figure 1:**
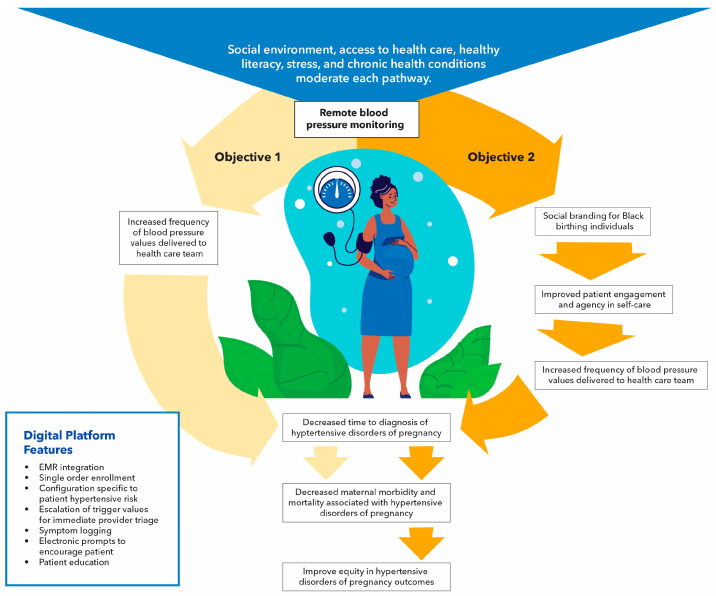
RBPM is expected to improve clinical management and individual outcomes for all patients; cultural tailoring to promote social identification mediates this pathway to achieve more equitable outcomes.

Figure 1 presents our conceptual framework and study objectives. We hypothesize that providing patients with RBPM through the selected technology will moderate prenatal care effects on the time to diagnosis of HDP, reducing maternal morbidity and mortality associated with the condition. Social and environmental variables, including stress, will moderate the effects. We further hypothesize that introducing cultural tailoring will mediate the desirability of engagement with RBPM, and that adherence to care plans will promote greater equity among users. 

### 2.2. Intervention Selection and Features

**Figure 2:**
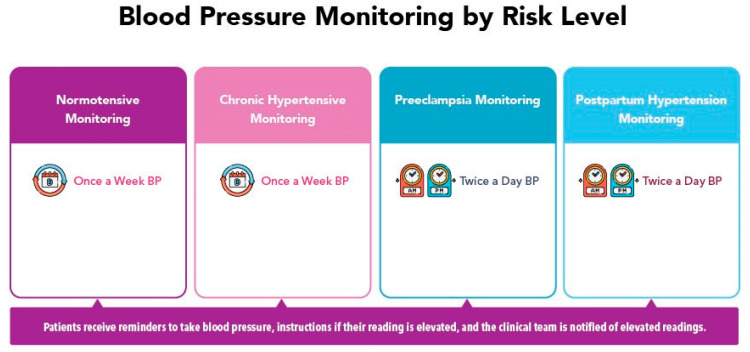
Frequency of blood pressure monitoring is specific to the diagnosis of each patient and reminders are sent to aid adherence to clinical management guidance.

Multiple options for RBPM and pregnancy applications exist. Our team selected this platform because it aligned with several project goals. For instance, it offers integration with the EHR and the use of an internet-enabled, electronic blood pressure cuff that automatically populates values, reducing the patient burden, and expediting access to blood pressure values. Integration of the digital platform with our EHR also facilitates seamless enrollment without an additional strain on staff resources. Enrollment occurs by placing a single order from the EHR, making it as easy to connect the patient to the platform as any other clinical order related to patient care. The selected platform also offers continuous monitoring of RBPM values (twenty-four hours a day, 365 days a year) and allows providers to select blood pressure targets aligned with the individual patient risk status. Figure 2 defines blood pressure targets by risk status. 

**Figure 3:**
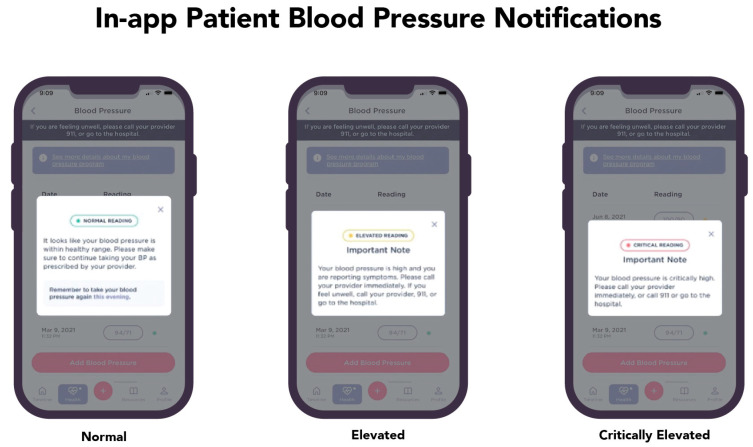
Users receive instructions as follow-ups to blood pressure readings and the clinical team is notified of abnormal readings in real-time.

Abnormal lab values are relayed to the physician or midwife for immediate triage. This feature delivers blood pressure readings outside identified ranges for the patient’s risk profile to the healthcare team, allowing for real-time intervention. Figure 3 illustrates the communication sent to users with abnormal blood pressure readings, which are automatically sent to the healthcare team for follow-up. 

The educational content is inclusive of a range of colors and textures for skin and hair, and includes social identities that are underrepresented in electronic applications and avatars. Furthermore, the written content offers the ability to adjust for different reading levels and avoid confusing idioms. This feature was essential to our selection as we serve many patients with low reading levels and use of English as a non-dominant language. Additionally, the media offers closed-caption capabilities, and an option to access the content in Spanish is available. Importantly, these flexibilities are extended to in-app text messaging sent directly to patients. were important, the ability to further customize content facilitates cultural tailoring and supports objective two.

### 2.3. Planned Analysis: Time to Diagnosis of Hypertensive Disorders of Pregnancy

A planned analysis will be conducted, with the aim of completing the research objectives, sequentially in two phases. The first research objective will be completed as phase 1 using a time-to-event strategy and intent to treat. In this phase, we will evaluate the days to diagnosis of HDP when using the digital platform, with an active comparison group. The comparison group will receive a digital platform that offers educational content but not RBPM. Patients will be assigned 1:1 using a random number generator to either the intervention (digital platform plus RBPM) or active comparison (digital platform). The hypotheses are the following:

1. Diagnosis of previously unrecognized chronic hypertension will increase with use of RBPM.
2. RPBM and use of the digital platform will decrease the days to diagnosis of HDP.
3. RBPM will decrease the time to diagnosis for all users regardless of demographic characteristics.

When phase 1 is completed, our team will incorporate culturally responsive social identification strategies to increase the engagement among Black patients. The content for cultural tailoring will be developed during phase 1 under a separate protocol not described here. The second research objective will be achieved by evaluating this tailoring in a randomized controlled trial as a second phase. The hypotheses are the following:

1. Cultural tailoring will promote social identification and mediate treatment effects on the time to diagnosis of HDP among Black users, narrowing the equity gap in outcomes.
2. Cultural tailoring will improve the perceived quality of care, reduce stress, and improve social support. These benefits will result in additional improvements in perinatal outcomes, such as fewer preterm birth and depression symptoms.

We further anticipate that social identification will create a consistent “brand” for the program (common desirable features, consistent imagery, look and feel) and will affect the time to diagnosis of HDP outcomes [38]. Recognition of the intervention and its tailored features will promote engagement and participation.

### 2.4. Outcome Measures

The planned primary outcome is the time to diagnosis of HDP. Planned secondary outcomes include an improvement in the perceived quality of care and reduced maternal stress. Additionally, we will measure the effect of cultural tailoring and its associated impact on health equity. While this study is not powered to detect differences in the rates of severe maternal morbidity, we will evaluate this outcome as an investigational hypothesis. 

We have selected biometric, behavioral, and social identification measures that are linked to our conceptual framework and hypotheses. EHR review will provide information on the diagnosis and timing, gestational age at delivery, mode of delivery, maternal comorbidities, and participation in postpartum care. The study instruments will be applied after consent and before enrollment, during pregnancy as specified, and at six to eight postpartum.

Blood pressure. Blood pressure will be taken using the internet-enabled blood pressure equipment provided, which will be linked to the digital platform and integrated with the EHR. These devices are calibrated and tested for consistency with hospital-based medical equipment. All recorded values will be included from the first prenatal visit to 8 weeks postpartum for research objectives 1 and 2. 

Laboratory values. The results of laboratory testing used to diagnose preeclampsia will be extracted from the EHR, including the complete metabolic panel, urine protein–creatinine ratio, and other data for research objectives 1 and 2. 

Quality of Prenatal Care Questionnaire [39]. The QPCQ is a reliable and validated instrument developed to assess the perceived quality of prenatal care. Reported Cronbach’s alpha = 0.96 and inter-item correlation coefficient = 0.88. This will be completed once each trimester for research objectives 1 and 2. 

Perceived Prenatal Maternal Stress Scale (PPNMSS). This validated instrument consists of 28 items organized as four sub-scales of perceived social support, pregnancy-specific concerns, intimate partner interaction, and economic matters [40]. Cronbach’s alpha = 0.83, with an inter-item correlation coefficient of <0.9. For research objectives 1 and 2, the PPNMSS will be completed once each trimester. 

Social identification. Brand equity is a term used to broadly define attitudes, perceptions, and connection that users attribute to a particular item or product. It has been successfully validated using a 16-item Likert scale reflecting four core constructs (loyalty, popularity, awareness, personality) as part of anti-smoking social marketing campaigns [41]. This scale will be adapted to measure social identification as part of research objective 2 and completed in the third trimester. 

Edinburgh Postnatal Depression Scale (EPDS). The EPDS is a validated 10-item screening assessment for depression symptoms during the perinatal period [42]. It also contains a sub-scale for anxiety. This scale has been proven to be highly sensitive and specific to both depression and change in depression over time (83.8% and 74.7%, respectively). For research objectives 1 and 2, all EPDS scores will be sourced from the medical record. The health system protocol is to complete the EPDS at each prenatal and postpartum visit. 

Uptake and Exposure. The digital platform will capture data on all aspects of user engagement, including content accessed by gestational age and postpartum stage, frequency of use, and duration (time) of each user session. These detailed usage data measure exposure and uptake for both research objectives. 

Process measures. The time from enrollment, first user activity, and number of RBPM entries will be included as measures for research objectives 1 and 2. 

### 2.5. Sample Size and Recruitment

The sample size was calculated using PROC POWER in SAS for a two-sided Score test in the Cox proportional hazard regression of the time to diagnosis of an HDP in the study group, with a binary variable taking on the values of intervention (digital platform plus RBPM) or active comparison (digital platform). The proportion of total participants in the intervention group was set at 50%. A hazard ratio of 1.4 for the study group was used as the effect size. The overall probability that a participant would be diagnosed with an HPD was set at 15.9% [43]. The total number of subjects needed to achieve a power of 80% with a significance level of 0.05 was determined to be 1745. 

Overall, HDP affects 15.9% of delivery hospitalizations in the US [43], and the prevalence is higher among patients served by the study site. In 24 months, our system provides prenatal care to over 3000 patients. Given this patient volume and prevalence, we considered the estimated sample size feasible with a 60% enrollment rate, which is consistent with the engagement with prior research studies in our setting of similar durations. We anticipate that the required cohort for each research objective will be enrolled within the desired timeline. The total active participant enrollment time for the planned protocol will be a total of 48 months: 24 months for phase 1, and 24 months for phase 2. 

All people presenting at the study site for prenatal care will be invited to participate at their initial prenatal visit by a study research coordinator. Individuals younger than 18 years will be excluded. Over 35% of patients receiving prenatal care at our facility identify as Black, which will allow us to test for the desired sub-group effects related to social identification. Randomization will occur at the time of enrollment, and baseline measures will be completed. Participants will receive USD 25 at the time of enrollment and each time they complete study data collection (enrollment, and then each subsequent trimester and postpartum) in recognition of the additional time required to complete these study materials. Survey data will be compiled using REDcap 14.2.1, a software distributed by Vanderbilt University in Nashville, Tennessee, United States, for non-commercial secure survey data collection, at enrollment and the subsequent timepoints.

### 2.6. Analysis

**Figure 4:**
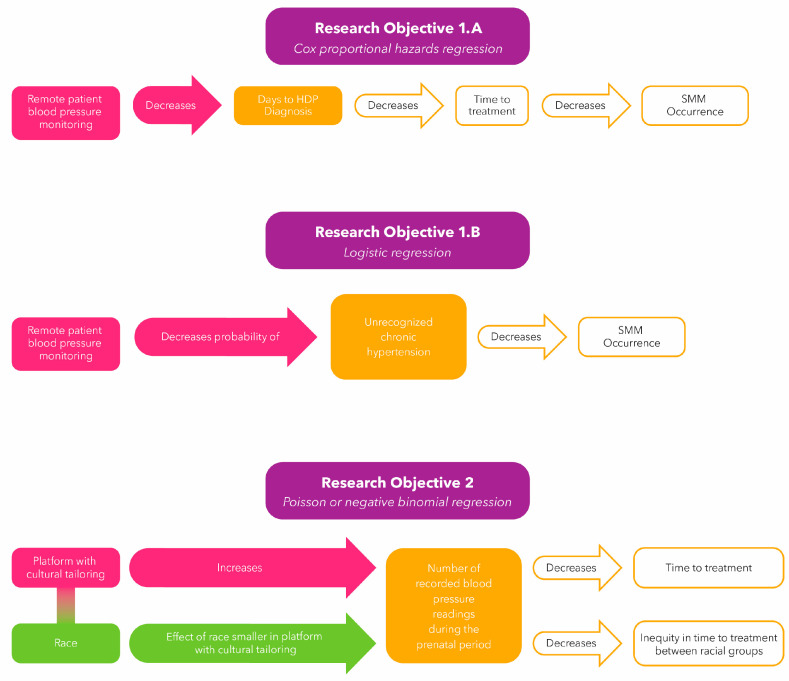
Three primary analyses are planned to accomplish research objectives 1 and 2.

The primary goal of the analysis will be to evaluate research objectives 1 and 2. For research objective 1, demographic data will be compared for the intervention and active comparison group. Time-to-event analysis will be used to compare the outcomes between these groups. We also will compare the total number and timing of blood pressure readings. For research objective 2, the analysis will focus on the mediation effects of social identification on health equity. Figure 4 illustrates the planned analyses for the primary outcome.

#### 2.6.1. Research Objective 1

##### Independent Variables and Covariates

The independent variable of interest will be a group variable. Participants will be randomized to one of two groups: the intervention group (digital platform with RBPM) or active comparison (digital platform, no RBPM). The covariates will be the age at enrollment in years, race, ethnicity, health insurance type (e.g., Medicaid, commercial, HMO), gestational age at first visit, and total number of prenatal visits. 

Demographic data will be summarized using means and standard deviations for continuous variables and frequencies and percentages for categorical variables. 

##### Objective 1.A and Analysis Plan

The primary outcome will be the days to diagnosis of an HDP, where an HDP is defined as gestational hypertension, preeclampsia, or preeclampsia with severe features. The days to diagnosis of an HDP will be calculated from the date of the first prenatal visit to the date of diagnosis (ICD-10-CM), clinical problem list (SNOMED-CT), and Intelligent Medical Objects (diagnosis and problems). A patient will be considered censored if they are never diagnosed with an HDP. A Cox proportional hazards regression will be used to model the effect of using the digital platform plus RBPM (intervention) compared to the digital platform alone (active comparison) on the days to HDP diagnosis. The regression coefficients and respective *p*-values, and the hazard ratios with confidence intervals will be reported. The predicted survival curves for each group will be plotted, along with the median days to diagnosis for each group. 

##### Objective 1.B and Analysis Plan

The second analyses will be a binary variable for having, or not having, a new diagnosis of chronic hypertension during the prenatal period prior to 20 weeks of gestation. A logistic regression will be used to model the probability of being diagnosed with a new case of chronic hypertension during the prenatal period by study group. Regression coefficients with their respective *p*-values, and odds ratios with confidence intervals will be reported. 

#### 2.6.2. Research Objective 2

##### Independent Variables and Covariates

The independent variable of interest for research objective 2 will be a group variable. Participants will be randomized to one of two groups: the intervention group (digital platform with RBPM and cultural tailoring) or comparison (digital platform with RBPM but no cultural tailoring). The covariates will be the age at enrollment in years, race, ethnicity, health insurance type (e.g., Medicaid, commercial, HMO), gestational age at first visit, and total number of prenatal visits. 

##### Objective 2 and Analysis Plan

The outcome, a count variable, will be defined as the total number of recorded blood pressure readings during the prenatal period. A Poisson or Negative Binomial regression will be used to model the number of recorded blood pressure readings during the prenatal period. Interactions between the study group and race will be tested. Significant interactions between a group and race would indicate that the effect of race differed between the study groups. If an interaction is statistically significant, predicted effects with 95% confidence intervals for race by study group will be provided. Regression coefficients with *p*-values from the model will be reported.

### 2.7. Data Approach

This study will use data both from the digital platform and the health system’s enterprise EHR. To support the cohort definitions for the randomized trial, digital platform users will be identified by a combination of referrals ordered through the Computerized Physician Order Entry (CPOE) system, matching of contributor systems (i.e., patients with RBPM integration), and industry-standard deterministic matching algorithms. Additionally, the team will use the ICD-10-CM (diagnoses), SNOMED-CT (problems), and Intelligent Medical Objects (diagnosis and problems) coding standards to identify the conditions of interest to support both the time to diagnosis outcome measurements. 

Digital platform data will consist of both manual and automatic data feeds, which will be matched using the approach outlined above. These RBPM readings will be aggregated, and user activity analysis will be aggregated and analyzed with temporal analysis using a multi-paradigm programming language. The number of blood pressure readings and user activity will be correlated with the information in the medical record to establish the relationship between engagement and outcomes. 

Many variables will be defined using EHR data recorded during perinatal care, including demographics, EPDS scores, utilization/engagement, and documentation from the delivery event. These heterogenous data elements are stored in the EHR as structured data to facilitate clean data extraction. They include diagnostics (e.g., lab orders), observations (e.g., vital signs), assessments (EPDS), and medical history (prior diagnoses and chronic conditions). 

## 3. Discussion and Conclusions

This planned protocol seeks to reduce maternal health inequities that cause disproportionate harm to Black women, birthing persons, and infants. In this work, we acknowledge the distinguishing qualities of “equity” efforts in comparison to “equality” efforts. While maternal morbidity and mortality impact women and birthing persons of all backgrounds, advancements in this field that center on those that have faced the greatest historical harm offer the greatest opportunity for a public health impact. 

Innovative features of the proposed digital platform expected to decrease the time to diagnosis of HDP include the integration with health system technology and patient records, blood pressure targets aligned with the individual patient risk status, and immediate triage of elevated blood pressure values to the clinical team for real-time intervention. Benefits for all patients are anticipated with the adoption of this intervention. The selected technology also allows for customization of the user-facing content. Leveraging this feature to offer cultural tailoring is expected to facilitate social identification and result in equity gains. Additional benefits are anticipated in the adjacent areas of quality and stress reduction, which may drive improvements in other clinical outcomes beyond the management of HDP. Further strengths of the analysis include its measures of patient exposure. 

The use of cultural tailoring to create social identification with the intervention and its contents and benefits is an important component of the planned protocol. This builds on prior work on healthy weight among the investigative team on promoting a healthy weight, which has shown promising early results in promoting participation and the use of digital intervention content [37].

Our evaluation of this intervention will contribute to the growing literature on digital health interventions to improve maternity care in the US. Furthermore, we propose a specific enhancement expected to promote equity and narrow the gap in outcomes experienced by historically marginalized populations. The proposed approach may be applicable to other efforts to test the specific benefits of cultural tailoring and social identification for public health interventions.

## Data Availability

No new data were created or analyzed in this study. Data sharing is not applicable to this article.
